# Carotid Artery Stenting in Patients with Symptomatic and Asymptomatic Stenosis: In-Hospital Clinical Outcomes at a Single Neurovascular Center

**DOI:** 10.3390/jcm11082086

**Published:** 2022-04-07

**Authors:** Kamran Hajiyev, Victoria Hellstern, Alexandru Cimpoca, Christina Wendl, Hansjörg Bäzner, Hans Henkes, Philipp von Gottberg

**Affiliations:** 1 Neuroradiologische Klinik, Klinikum Stuttgart, D-70174 Stuttgart, Germany; v.hellstern@klinikum-stuttgart.de (V.H.); a.cimpoca@klinikum-stuttgart.de (A.C.); hhhenkes@aol.com (H.H.); p.vongottberg@klinikum-stuttgart.de (P.v.G.); 2 Institut für Röntgendiagnostik, Zentrum für Neuroradiologie, Fakultät für Medizin, Universität Regensburg, D-93053 Regensburg, Germany; christina.wendl@ukr.de; 3 Neurologische Klinik, Klinikum Stuttgart, D-70174 Stuttgart, Germany; h.baezner@klinikum-stuttgart.de; 4 Medizinische Fakultät, University Duisburg-Essen, D-47057 Duisburg, Germany

**Keywords:** carotid artery stenosis, neurointervention, carotid artery stenting, endovascular treatment, stroke

## Abstract

Background: Carotid artery stenting (CAS) is a minimally invasive and proven percutaneous procedure that is widely used to treat patients with symptomatic and asymptomatic carotid artery stenosis. The purpose of this study was to characterize the in-hospital outcomes of symptomatic and asymptomatic patients undergoing CAS at a single neurovascular center. Methods: The study was conducted as a retrospective analysis of 1158 patients (asymptomatic, *n* = 636; symptomatic, *n* = 522; male, *n* = 816; median age, 71 years; NASCET method, 70–99% stenosis, *n* = 830) who underwent CAS between May 2009 and December 2020. In-hospital neurological outcomes, adverse reactions to iodinated contrast media, acute myocardial infarction, intraprocedural complications, and access-site issues were evaluated. The primary endpoints were disabling stroke (including disabling cerebral hyperperfusion syndrome [CHS]) and all in-hospital deaths. Results: A carotid stent could not be deployed in one patient due to calcified plaques (technical failure rate of 0.09%). Four patients (0.3%) experienced in-hospital, stroke-associated death, while five patients (0.4%) died from non-stroke-related causes. All stroke-associated deaths occurred in the symptomatic group and were due to CHS. The disabling stroke rate was 0.9% overall (*n* = 10; 0.5% versus 1.3% in asymptomatic versus symptomatic patients, respectively). Nineteen patients (1.6%) reached the in-hospital primary endpoint. More patients in the symptomatic group achieved this endpoint than in the asymptomatic group (2.5% versus 0.9%, respectively; *p* = 0.060). Conclusions: An evaluation was conducted on the in-hospital outcomes of 1158 patients at a single center who underwent CAS and was performed by trained physicians who were supervised by a senior neurovascular interventionist with over 20 years of experience, confirming the excellent safety profile of this procedure with a low rate of complications.

## 1. Introduction

Since the publication of the first results from the North American Symptomatic Carotid Endarterectomy Trial (NASCET) over 30 years ago, several major trials have confirmed the beneficial impact of carotid endarterectomy (CEA) for the prevention of major ipsilateral strokes in patients with symptomatic or high-grade proximal internal carotid artery (ICA) stenosis [1,2,3,4]. Thus, international guidelines currently suggest CEA as the first-line, non-conservative treatment of symptomatic or high-grade ICA-stenosis [5,6,7]. Carotid artery stenting (CAS) is presented as an alternative treatment for a subset of selected patients. This is primarily due to the reported higher risk of periprocedural ipsilateral minor infarction in patients above the age of 70 years [8,9,10].

However, the risks of ipsilateral stroke secondary to CAS vary. Results are typically reported in studies that combine data from different medical centers with varying levels of experience and expertise with this procedure. Short- and long-term outcomes of both CAS and CEA reported in monocentric studies are frequently similar to one another [11,12]. Careful selection and preparation of patients for CAS may further reduce the risk of periprocedural complications [13,14,15].

This study was conducted to reassess the safety of CAS using data from a high-volume neurointerventional center and to evaluate periprocedural risks compared with those reported in previous studies.

## 2. Materials and Methods

The monocentric retrospective analysis was approved by the local ethics committee (IRB number: F-2018-085). Written informed consent was obtained from all patients before undergoing the procedure. Demographic, clinical, and periprocedural data were collected retrospectively. Post-procedural primary and secondary endpoints were analyzed statistically.

### 2.1. Patient Selection and Evaluation

All carotid artery stenoses were assessed by duplex ultrasound, computed tomography (CT)-angiography, or magnetic resonance imaging (MRI)-angiography and confirmed by digital subtraction angiography (DSA). Inclusion criteria were:

- patients diagnosed with atherosclerotic proximal ICA stenosis.
- no limit on patient age.
- symptomatic patients with no limit on the degree of stenosis or preprocedural modified Rankin Scale (mRS) score.
- asymptomatic patients with ICA stenosis >50% or <50% stenosis with vulnerable plaques (i.e., plaque-sealing). A plaque was considered vulnerable if its contour or surface was extraordinarily irregular or ulcerated and, thus, if the plaque was likely to have caused the most recent neurologic events.
- patients with radiation-induced stenosis.
- patients with post-CEA re-stenosis.

Patients with ICA dissection, tandem occlusion associated with acute ischemic stroke, extra- and intracranial stenoses that both require treatment, stenosis due to neck tumors, acute intracranial hemorrhage, and/or contraindications for dual antiplatelet therapy were excluded.

The pre-procedural evaluation included a neurological assessment (National Institutes of Health Stroke Scale [NIHSS] and mRS [16,17]), an assessment of the degree of stenosis, laboratory results, Multiplate and VerifyNow^®^ tests (for patients treated after 2012). Stenosis was considered symptomatic if the patient experienced transient ischemic attacks (TIAs), amaurosis fugax, or chronic hemodynamic compromise and cerebral infarction of the corresponding ICA territory in the preceding six months or acute cerebral ischemia in the last seven days. Patients were considered asymptomatic if they had neither stroke nor TIAs (hemispheric or ocular) within the preceding six months (Table 1). The NASCET method was used to determine the degree of stenosis. We have been using the Multiplate and VerifyNow^®^ tests since 2012 to monitor each patient’s response to antiplatelet drugs and to select a suitable dosage and drug combination. Loading doses of acetylsalicylic acid (500 mg [intravenous] IV) and clopidogrel (600 mg per os [PO]) or ticagrelor (180 mg PO) were administered in emergency cases. In non-emergency cases, patients who were not on anticoagulation therapy received daily dual antiplatelet therapy for at least three days before the procedure. Patients managed with long-term anti-aggregation were also examined with Multiplate and VerifyNow^®^ tests to evaluate platelet dysfunction and, if necessary, to provide treatment with another type of anti-aggregation drug.

Baseline demographics, risk factors, and anatomical data were evaluated in all patients. These findings are summarized in Table 2, Table 3 and Table 4.

### 2.2. Intervention Protocol and Technical Data

All CAS procedures were performed by trained neurointerventionalists with more than three years of experience with endovascular techniques. These physicians were closely supervised by the senior author who has over 20 years of experience with neuroendovascular procedures. The interventional techniques and materials have been refined over the years and include different types of stents and balloons (Table 5). The procedural standards for a stent-assisted ICA angioplasty in our institution for the patients evaluated in this study are described in the paragraphs to follow.

Heart rate and arterial blood pressure were monitored continuously during the procedure. Selective catheterization of the common carotid artery (CCA) was typically performed using an 8F guiding catheter (Guider Softip^TM^; Stryker, Kalamazoo, MI, USA). Diagnostic angiography was performed to confirm the precise degree of stenosis and to evaluate the extent of collateralization. Unfractionated heparin was infused as a bolus of 3000 to 5000 IU. An embolic protection device (EPD) was used in only 39 (3.4%) of the patients. A 0.014-inch microguidewire (e.g., Hi-Torque All Star 0.014″; Abbott, Chicago, IL, USA) was gently inserted into the cervical ICA. Before balloon dilatation, a bolus of 0.5–1 mg of atropine was administered intravenously to prevent bradycardia or cardiac standstill due to vasovagal reflex.

The region of stenosis was pre-dilated using a 4 mm non-compliant balloon (e.g., pITA 4/40; phenox). A stent was then inserted and deployed. Post-dilatation of the stent was performed in selected cases. A final angiogram of the cerebral circulation was performed to confirm the correct positioning of the stent and to rule out distal emboli. Residual stenosis <50% with no alteration of the brain circulation is considered a satisfactory result. A vascular closure device (AngioSeal^®^, Terumo, Shibuya City, Tokyo, Japan) was preferred for the final repair of the access site. However, in 94 patients, the arterial access site was closed by manual compression followed by a 24 h compression bandage alone.

### 2.3. Post-Procedural Period

After the procedure, all patients underwent an in-hospital neurological assessment and, in most cases, post-procedural brain imaging with CT or MRI before the hospital discharge (*n* = 880). MRI was carried out following our standard-of-care protocol (*n* = 776). A brain CT scan, or in very few cases no brain imaging, was obtained only in patients with contraindications to MRI. Images were read carefully for signs of acute of subacute infarction, i.e., DWI-lesions and new cortical hypodensities, as well as ICB.

Arterial blood pressure was monitored throughout and maintained at a systolic level of 120–130 mmHg. IV hydration was administered to patients who developed contrast-induced nephropathy (CIN). In-hospital neurological events were documented and categorized as follows:

- Transient ischemic attack (TIA)—a reversible focal neurological deficit of short duration (<3 h) without stroke.
- Cerebral hyperperfusion syndrome (CHS)—diagnosed based on clinical symptoms that range from headaches to seizures and focal neurological defects associated with brain edema and intracerebral or subarachnoid hemorrhage. While CHS has been linked to impaired cerebrovascular autoregulation [18], the underlying pathophysiology of this syndrome is not yet fully understood.
- Stroke—defined as an acute focal neurological deficit with cerebral ischemia and categorized as
- Non-disabling—an increase on the modified Rankin Scale (mRS) score of ≤2 points from pre-stroke status, or
- Disabling—an increase on the mRS of 3 points or more.
- A creatine kinase (CK)-defined myocardial infarction—CK-myocardial band (MB) or troponin levels that were greater or equal to two times the upper limit of the normal range, together with chest pain, symptoms consistent with ischemia, and/or development of specific abnormalities on a standard 12-lead electrocardiogram.

The primary endpoint of this study was disabling stroke (including CHS) and all in-hospital deaths. Primary technical success rate, in-hospital neurological and adverse events, CHS, acute myocardial infarction, CIN, as well as intraprocedural and access-site complications were assessed. The rate of restenosis and in-stent thrombosis were also evaluated.

Patients were discharged on dual antiplatelet therapy that included aspirin (100 mg/day PO) with clopidogrel (1 × 75 mg/day PO), ticagrelor (2 × 90 mg/day PO), or prasugrel (1 × 10 mg/day PO).

### 2.4. Statistical Analysis

Numbers and percentages were used to describe categorical data; median, minimum, and maximum were shown for continuous variables. Fisher’s exact test was used to determine independence between two categorical variables. The Mann–Whitney U test was used to determine whether the distribution of continuous variables was the same in the two groups. All statistical tests were two-sided, with a level of significance set to 0.05. Odds ratios (ORs) with 95% confidence intervals (CIs) were calculated to quantify the association between a variable and an outcome. Statistical analyses were performed using Stata/IC 16.1 for Unix (StataCorp 4905 Lakeway Drive, College Station, TX 77845, USA).

## 3. Results

A total of 1248 CAS procedures were performed in 1158 patients at a single neurovascular center between May 2009 and December 2020. The full patient cohort included 342 females and 816 males at a median age of 71 years (range 41–96 years). The most common comorbidities were arterial hypertension (82.6%), dyslipidemia (50%), coronary artery disease (38.9%), and diabetes mellitus (34.7%). Forty-four patients (3.8%) were diagnosed with carotid artery stenosis secondary to previous neck radiation. Seventy-one patients (6.1%) presented with restenosis after a previous CEA. Forty-six patients (4%) exhibited contralateral ICA stenosis of >75%; 136 patients (11.7%) were diagnosed with contralateral ICA occlusion. We identified 830 patients (71.7%) with severe stenosis (NASCET determination, 70–99%).

The common femoral artery was punctured under local (*n* = 222) or general (*n* = 936) anesthesia, and an 8F femoral sheath was placed. In three cases, the brachial artery was chosen due to the severe occlusive disease of the pelvic arteries. The stent was pre-dilated in 1103 patients (95.3%); additional post-dilatation was performed in 134 of these cases (11.6%). In 44 patients (3.8%), the stent was placed with no pre-or post-dilatation (i.e., “plaque sealing”). Open- or closed-cell stents were used in 197 (17%) and 961 (83%) of the procedures, respectively (Table 5). The 7/40 Carotid Wallstent (Boston Scientific) was selected for >80% of these procedures. An additional stent was required in 43 patients (3.7%) for better plaque coverage. In one patient (0.09%), the carotid stent could not be deployed due to calcified plaques. The stent was accidentally deployed in one other case that resulted in an iatrogenic dissection. The median intervention time was 33 min, and the median x-ray exposure time was 19.1 min. Eleven patients (0.9%) exhibited residual stenosis of >50%, which was re-diluted in a later session. The median hospitalization time was five days, although this period was significantly longer in the symptomatic group (*p* < 0.001).

### 3.1. Intraprocedural Complications

Nine patients (0.8%) experienced intraprocedural complications; these were more frequent in the symptomatic group (*n* = 3 versus *n* = 6). Among these complications:

- Five patients developed ICA dissection.
- Three patients were diagnosed with distal embolization as documented in the final angiogram.
- One patient developed perforation of a side branch of the external carotid artery due to unexpected and excessive wire relaxation. The lesion was glue-embolized immediately.
- While two patients exhibited TIAs, new-onset cerebral ischemia was not observed.

### 3.2. Primary Endpoints

The primary composite endpoint was reached by 1.6% of the patients overall and at a higher rate in the symptomatic group (0.9% versus 2.5%, *p* = 0.060; Table 6).

Further details of these endpoints include:

- Ten patients developed disabling strokes.
- Four patients died while in the hospital. All four were stroke-associated deaths among patients in the symptomatic group. The deaths were the result of severe bleeding associated with CHS.
- The five non-stroke deaths resulted from sepsis secondary to respiratory or urogenital infections; one patient died as a result of spontaneous cardiac arrest.

### 3.3. Secondary Endpoints

Twenty-three patients (2%) developed non-disabling strokes. Reversible adverse reactions to iodinated contrast media (ICM) and TIAs were observed in another 11 patients (0.9%). Twenty-five patients developed CHS (2.2%) within 24 h after the CAS procedure. Seventeen of these patients exhibited no permanent neurological deficits on clinical examination by a consulting neurologist upon discharge from our hospital (2–79 days, median 12 days) and had no recurrence of symptoms within 30 days following the procedure. Five of these patients developed disabling strokes and three developed non-disabling strokes. Three additional patients (0.3%) who presented with histories of coronary artery disease experienced acute myocardial infarction. Fifteen patients (1.3%) developed acute CIN or contrast agent-associated nephropathy; seven of these patients had histories of chronic renal insufficiency (*p* = 0.001). Seventy-eight patients (6.7%) experienced access-site complications; 26 of these patients developed femoral pseudoaneurysm, and 11 developed femoral artery occlusions.

During a median follow-up of eight months, 101 patients developed in-stent restenosis (8.7%; *n* = 26 >75%) or in-stent thrombosis (1.2%; *n* =14). Eighty-three of these patients required follow-up treatment.

Apart from endpoints, 776 patients underwent MRI examinations following CAS that revealed silent DWI-microlesions in 360 patients (29.7% vs. 32.8% asymptomatic and symptomatic; 31.7% vs. 27.9% closed-cell and open-cell stents, respectively).

## 4. Discussion

Extracranial ICA stenosis is a common disease in numerous populations worldwide. Earlier estimates suggest that this disorder will eventually occur in 4–7% of middle-aged and older adults [19]. ICA stenosis represents a major component of the estimated 7–15% of strokes directly attributable to cerebrovascular disease [20,21,22,23]. The main risk factors associated with this condition include arterial hypertension, diabetes, hyperlipidemia, and chronic coronary vessel disease, with a clear male predominance [19,24].

The 1158 patients with either symptomatic or asymptomatic ICA stenosis described in this study presented with major risk factors that were comparable and distributed as described in several large studies that were published previously. The rates of periprocedural (i.e., <30 days post-procedure) stroke and death reported here were lower, albeit similar to those reported by major randomized controlled multicenter trials that compared the outcomes of CAS versus CEA, including CREST [9], ACT 1 [25], SAPPHIRE [26], and SPACE-2 interim analysis [27]. Our findings were surpassed only in one respect by the data reported from the ACST-2 trial [4] (Table 7), suggesting that there may be untapped potential in improving patient outcomes.

One possible explanation for this observation may relate to the lack of randomization in our study. Our study included only patients who had been selected to undergo CAS; patients in the earlier trials were randomly assigned to this procedure. However, our criteria for CAS and patient profiles were comparable to those in the aforementioned studies. This suggests that there was unlikely to be any significant difference in the risk of developing complications.

The findings presented in this study include data from patients with ICA stenosis graded <60% (NASCET method) who underwent CAS to treat asymptomatic, presumably ruptured plaque (i.e., “plaque sealing”). No pre- or post-stent dilation was performed in these patients to reduce the risk of plaque debris embolism, vasospasm, and/or ICA dissection. However, these patients represent a very small percentage of the entire cohort (*n* = 44, 3.8%), and their rate of periprocedural complications is comparable to that of the stenosis group (*n* = 1, 2.2%).

The rate of periprocedural minor/non-disabling stroke determined for our patient population was lower than those reported in multicenter randomized trials. Of note, the CAS technique used here does not differ significantly from those described in these studies.

The details of the CAS procedures performed in this study reflect the frequently-described benefits of closed versus open-cell stents [28] whenever possible. Our findings are fairly consistent with the advantages associated with the use of closed-cell stents (i.e., lower rates of plaque debris embolism). In our study, the incidence of disabling and non-disabling stroke was nearly two times higher in patients who underwent CAS with an open rather than a closed-cell stent (4.6% among those undergoing open-cell CAS versus 2.5% for those undergoing closed-cell CAS). This is particularly notable given that the number of cases involving open-cell stents is approximately five times smaller (*n* = 197 cases with open-cell stents versus *n* = 961 cases with closed-cell stents).

In comparison to the above-mentioned trials, as far as verifiable, our number of deployed open-cell stents is fairly comparable (30% in ACST-2 [4]) or even higher (e.g., closed-cell stents exclusively in ACT-1 [25]).

The CAS procedure used in this study favors pre-dilation versus post-dilation of the stenosis and generally attempts to avoid more than one dilation [29]. Furthermore, a balloon size of 4 mm was carefully chosen as standard in the CAS procedures at our hospital to avoid overstretching of plaque and the vessel.

Among the patients with access site complications, 37 (3.2%) needed further treatment through vascular surgery. Changing the standard approach from transfemoral to transbrachial with the intention to reduce access site complications has been a topic of internal discussion in our institution; however, recent studies found no significant difference in the rate of these complications when comparing the transfemoral and transbrachial approaches [30,31,32]. In addition, the use of an 8F guide catheter for CAS, as is widely used, does not enable transradial access.

The patient collective, selection criteria for CAS and the CAS procedure do not differ significantly from those of the mentioned multicenter trials. The CAS procedure in particular does not differ from internationally accepted and well described CAS procedures. The high case number at our institution may be the most prominent difference.

The terms for qualification as a center in trials, such as ACST, ACT-1, and ACST-2, required participants to have carried out at least 25 CAS procedures over the last years of a stroke and death risk less than or equal to 8% for symptomatic, and 4% for asymptomatic ICA stenosis patients [4,25,33]. In SAPPHIRE, an incidence of periprocedural stroke and death of less than 6% and an overall treatment experience of at least 20 CAS procedures (median *n* = 64) was accepted for qualifying as a center [26]. SPACE-2 neurointerventionalists were eligible if they had carried out 40 CAS in general and 20 CAS within SPACE-1 with a peri-interventional complication rate under 6% [34].

In 2009, at the beginning of our retrospective data collection, the senior neurointerventionalist supervising all the procedures that were ultimately included in the database had a full 19 years of personal experience with neurointervention techniques and had personally performed >1000 CAS procedures, surpassing by far the highest reported amount of CAS procedures of any participant in the mentioned trials (SAPPHIRE, *n* = 700 [26]). Additionally, the high amount of neurointerventional procedures at our facility provided regular theoretical and practical training of anesthesiologists and the medical-technical staff in neurointerventional treatment. This aspect is crucial and continues today. The authors of this study are aware that multicenter trial data and our institutions’ data are not directly comparable.

Nevertheless, it is possible that the low rates of periprocedural deaths, as well as major and minor strokes, may relate to the fact that the study was performed at a single center with high case numbers. In this setting, all medical personnel may gain substantial relevant experience. This critical experience, combined with the contributions of specialized staff and equipment, may create a setting in which neuro-interventional patient care is both highly effective and sustainable.

## 5. Conclusions

The findings presented in this study document a lower rate of periprocedural stroke and death associated with CAS procedures from a single, high caseload neurointerventional center compared to the rates reported in earlier multicenter trials. Our results may suggest that single large centers may serve as a source of critical experience as well as the specialized personnel, equipment, and infrastructure needed to carry out neurointerventions in general and CAS in particular with high rates of safety and success.

## Figures and Tables

**Table 1:** Patient classifications according to clinical symptoms and brain imaging.

		*n* (%)
Symptomatic	Total	522 (45.1%)
	Acute ipsilateral stroke during the last 7 days	214 (18.5%)
	Chronic hemodynamic ischemia	141 (12.2%)
	TIA *	111 (9.6%)
	Amaurosis fugax	56 (4.8%)
Asymptomatic	Total	636 (54.9%)

* transient ischemic attack.

**Table 2:** Risk factors.

		Total (*n* = 1158)	Asymptomatic (*n* = 636)	Symptomatic (*n* = 522)	*p*
Gender	Female	342 (29.5%)	187 (29.4%)	155 (29.7%)	0.948 *
	Male	816 (70.5%)	449 (70.6%)	367 (70.3%)	
Age (years)	Median	71	71	72	0.809 **
	Range	41–96	46–94	41–96	
	≥80 (*n*, %)	193 (16.7%)	91 (14.3%)	102 (19.5%)	
Atrial fibrillation		126 (10.9%)	72 (11.3%)	54 (10.3%)	0.636 *
Diabetes mellitus		402 (34.7%)	195 (30.7%)	207 (39.7%)	0.002 *
History of tobacco use		284 (24.5%)	145 (22.8%)	139 (26.6%)	0.149 *
Arterial hypertension		957 (82.6%)	521 (81.9%)	436 (83.5%)	0.484 *
Peripheral artery disease		235 (20.3%)	137 (21.5%)	98 (18.8%)	0.271 *
Coronary artery disease		450 (38.9%)	284 (44.7%)	166 (31.8%)	<0.001 *
History of myocardial infarction		223 (19.3%)	139 (21.9%)	84 (16.1%)	0.014 *
Cardiac pacemaker		54 (4.7%)	36 (5.7%)	18 (3.4%)	0.092 *
Chronic renal insufficiency		149 (12.9%)	77 (12.1%)	72 (13.8%)	0.428 *
Fibromuscular dysplasia		6 (0.5%)	4 (0.6%)	2 (0.4%)	0.696 *
Dyslipidemia	Hypercholesterolemia	350 (30.2%)	193 (30.3%)	157 (30.1%)	0.037 ^#^
	Hypertriglyceridemia	12 (1%)	3 (0.5%)	9 (1.7%)	
	Hyperlipoproteinemia	114 (9.8%)	54 (8.5%)	60 (11.5%)	
	Combined dyslipidemia	103 (8.9%)	51 (8%)	52 (10%)	
Body mass index, (BMI) kg/m^2^ (median, range)		26 (15–47)	26 (15–43)	26 (16–47)	0.235 *
Previous neck radiation		44 (3.8%)	24 (3.8%)	20 (3.8%)	1.000 *
Previous CEA ***		71 (6.1%)	51 (8%)	20 (3.8%)	0.003 *

* Fisher’s exact tests; ** Mann–Whitney U test; ^#^ global test for independence between groups (asymptomatic/symptomatic) and type of the dyslipidemia; *** Carotid endarterectomy.

**Table 3:** Distribution of the risk factors among male and female patients.

	Total (*n* = 1158)	Female (*n* = 342)	Male (*n* = 816)
Age (median, range)	71, 41–96	71, 41–92	71.5, 44–96
Arterial hypertension	957 (82.6%)	274 (80.1%)	683 (83.7%)
Dyslipidemia (all)	579 (50%)	176 (51.5%)	403 (49.4%)
Diabetes mellitus	402 (34.7%)	114 (33.3%)	288 (35.3%)
Coronary artery disease	450 (38.9%)	110 (32.2%)	340 (41.7%)
Peripheral artery disease	235 (20.3%)	60 (17.5%)	175 (21.4%)

**Table 4:** Anatomical data.

		Total (*n* = 1158)	Asymptomatic (*n* = 636)	Symptomatic (*n* = 522)	*p*
Contralateral ICA stenosis	25–50%	104 (9%)	54 (8.5%)	50 (9.6%)	<0.001 ^#^
	50–75%	126 (10.9%)	63 (9.9%)	63 (12.1%)	
	>75%	46 (4%)	14 (2.2%)	32 (6.1%)	
	Stent	77 (6.6%)	61 (9.6%)	16 (3.1%)	
Contralateral ICA occlusion	acute	7 (0.6%)	5 (0.8%)	2 (0.4%)	0.659 ^#^
	chronic	129 (11.1%)	69 (10.8%)	60 (11.5%)	
Contralateral acute stroke		45 (3.9%)	27 (4.2%)	18 (3.4%)	0.543 *
Location of stenosis	right	635 (54.8%)	360 (56.6%)	275 (52.7%)	0.192 *
	left	523 (45.2%)	276 (43.4%)	247 (47.3%)	
NASCET ** (%)	<50%	19 (1.6%)	14 (2.2%)	5 (1%)	0.080 ^#^
	50–69%	309 (26.7%)	180 (28.3%)	129 (24.7%)	
	70–99%	830 (71.7%)	442 (69.5%)	388 (74.3%)	
Ulceration		507 (43.8%)	254 (39.9%)	253 (48.5%)	0.004 *
Pre/poststenotic dilatation	pre	62 (5.4%)	34 (5.3%)	28 (5.4%)	0.157 *
	post	302 (26.1%)	180 (28.3%)	122 (23.4%)	

* Fisher’s exact tests; ^#^ global test; ** North American Symptomatic Carotid Endarterectomy Trial.

**Table 5:** Stent type.

		Total (*n* = 1158)	Asymptomatic (*n* = 636)	Symptomatic (*n* = 522)	*p* *
Name of stent	CGuard™	28 (2.4%)	13 (2%)	15 (2.9%)	0.011
	CASPER™	2 (0.2%)	2 (0.3%)	0 (0%)	
	Cristallo™	91 (7.9%)	66 (10.4%)	25 (4.8%)	
	Gore^®^	8 (0.7%)	5 (0.8%)	3 (0.6%)	
	Herculink^®^	1 (0.1%)	1 (0.2%)	0 (0%)	
	Protégé™	69 (6%)	35 (5.5%)	34 (6.5%)	
	Wallstent™	955 (82.5%)	512 (80.5%)	443 (84.9%)	
	Xact^®^	4 (0.3%)	2 (0.3%)	2 (0.4%)	
Stent design	Closed-cell	961 (83%)	516 (81.1%)	445 (85.2%)	0.071
	Open-cell	197 (17%)	120 (18.9%)	77 (14.8%)	

* Fisher’s exact tests.

**Table 6:** Primary endpoints.

		Total (*n* = 1158)	Asymptomatic (*n* = 636)	Symptomatic (*n* = 522)	OR (95% CI)	*p* *
Disabling stroke		10 (0.9%)	3 (0.5%)	7 (1.3%)	2.87 (0.74–11.17)	0.124
Death	Stroke-associated deaths	4 (0.3%)	0 (0%)	4 (0.8%)		0.041
	Non-stroke deaths	5 (0.4%)	3 (0.5%)	2 (0.4%)		1.000
Disabling stroke and all deaths		19 (1.6%)	6 (0.9%)	13 (2.5%)	2.68 (1.01–7.12)	0.060

* Fisher’s exact tests.

**Table 7:** Comparison of periprocedural complication rate in different randomized multicenter trials.

Study Name	Total CAS *	Periprocedural Disabling Stroke and/or Death	Periprocedural Minor/Non-Disabling Stroke
CREST [9]	594	2.5% (*n* = 15)	2.0% (*n* = 12)
SAPPHIRE [26]	159	4.4% (*n* = 7)	3.1% (*n* = 5)
SPACE-2 Interim [27]	197	2.5% (*n* = 5)	6.6% (*n* = 13)
ACT 1 [25]	1072	2.9% (*n* = 31)	2.4% (*n* = 26)
ACST-2 [4]	1811	0.8% (*n* = 15)	2.7% (*n* = 48)
This study	1158	1.6% *(n* = 19)	2.0% (*n* = 23)

* Carotid artery stenting.
